# Moxibustion for the side effects of surgical therapy and chemotherapy in patients with gastric cancer: A protocol for systematic review and meta-analysis

**DOI:** 10.1097/MD.0000000000021087

**Published:** 2020-07-17

**Authors:** Shuqing Li, Jianrong Chen, Yanping Wang, Xu Zhou, Weifeng Zhu

**Affiliations:** a Evidence-based Medicine Research Center, Jiangxi University of Traditional Chinese Medicine, Jiangxi; b Second Clinical Medical College, Chongqing Medical University, Chongqing, China.

**Keywords:** gastric cancer, moxibustion, protocol, side effects, systematic review

## Abstract

**Background:**

Side effects after surgical therapy and chemotherapy of gastric cancer substantially reduce patients’ quality of life. This systematic review aims to investigate whether moxibustion, as a complementary treatment, is effective in alleviating side effects in patients with gastric cancer who underwent surgical therapy or chemotherapy.

**Methods:**

We will systematically search nine English and Chinese electronic databases to find relevant randomized controlled trials (RCTs) that compare basic treatment with and without moxibustion for treating the side effects induced by surgical therapy or chemotherapy in patients with gastric cancer. The time frame of the search will be from inception to July 1, 2020, and the publication language will not be limited. The literature screening and data extraction will be completed independently by 2 reviewers. The Cochrane risk of bias tool will be used to assess the risk of bias. For the analyses of the side effects of both surgical therapy and chemotherapy, the primary outcomes are defined as the incidence of any side effect, response rate, and quality of life. For the analyses of the side effects of surgical therapy, the secondary outcomes include the incidence of each individual side effect, time to first flatus/defecation/bowel sounds, and length of in-hospital stay. For the analysis of the side effects of chemotherapy, the secondary outcomes include incidence of each individual side effect, white blood cell/red blood cell/platelets counts, and hemoglobin level. R v3.6.2 software will be used to perform the meta-analyses. The quality of evidence will be classified using the Grading of Recommendations Assessment, Development and Evaluation system.

**Results:**

This study will provide the first systematic review evidence on the efficacy of moxibustion as adjuvant management for gastric cancer by rigorous quality assessment and appropriate data synthesis. The results will be submitted to a peer-reviewed journal for publication.

**Conclusion:**

The findings of this study will provide currently best evidence on moxibustion for patients with gastric cancer who underwent surgical therapy or chemotherapy and may impact clinical practice.

PROSPERO registration number: CRD42020169511

## 1 Introduction

Gastric cancer is a high-incidence malignant disease with a poor prognosis that poses a serious threat to global health.^[1]^ Worldwide, there were approximately 122,0000 new cases of gastric cancer and 865,000 deaths in 2017, accounting for 6.8% (fifth rank) and 8.8% (third rank) of the overall cancer incidence and mortality, respectively.^[2,3]^ China carries a burden of disease above the average level, where the official data reported that there were approximately 679,000 new cases of gastric cancer and 498,000 deaths in 2015.^[4]^

Routinely, surgical therapy and chemotherapy are the main therapies for gastric cancer and can greatly reduce the tumor load and prolong the survival.^[5]^ However, patients usually complain about the side effects following these therapies.^[6]^ For example, after surgical therapies, patients easily experience abdominal distension, constipation or diarrhea due to gastrointestinal reconstruction, anesthesia, and traction stimuli;^[7]^ moreover, many chemotherapy drugs can attack bone marrow and increase the level of 5-hydroxytryptamine_3_ released from gastrointestinal chromaffin cells, leading to myelosuppression and severe nausea and vomiting.^[8,9]^ These side effects substantially impede the acceptance of the therapies, reduce patients’ quality of life and even affect the anti-cancer efficacy.^[10]^ The current guidelines of Western medicine, however, do not provide specific treatments for the gastrointestinal side effects except for the suggestions of preoperative fasting, maintenance of electrolyte balance, and nutrition support, the effectiveness of which are limited.^[11]^ Therefore, the identification of new approaches to treat the gastrointestinal side effects after surgical therapy and chemotherapy is warranted in the management of gastric cancer.

Moxibustion, a traditional acupoint therapy depending on moxa-heat stimulation, has been widely used as a complementary treatment for various gastrointestinal disorders.^[12]^ For example, a systematic review of seven randomized controlled trials (RCTs) for irritable bowel syndrome found that the digestive symptoms, including abdominal pain, abdominal distension, and diarrhea symptoms, were significantly alleviated after two to four weeks of moxibustion.^[13]^ Another systematic review including patients with ulcerative colitis has also revealed that compared with drug therapy alone, moxibustion-assisted therapy significantly improved the response rate classified by the digestive symptoms and findings of endoscopy.^[14]^ From the mechanistic perspective, the moxibustion-heat stimulation of acupoints can promote the recovery of the gastrointestinal electrical rhythm along the meridians and collaterals, to adjust the gastrointestinal motility and relieve the postoperative gastrointestinal discomforts.^[15]^ Heat stimulation can also inhibit the release of 5-hydroxytryptamine_3_ and promote the generation of granulocyte colony stimulating factor, resulting in the prevention of nausea and vomiting and the proliferation of hematopoietic cells.^[16]^

Based on these previous findings, we hypothesized that moxibustion could be used to alleviate the side effects of gastric cancer treatments. To date, multiple RCTs assessing the hypothesis has been published but their findings are inconsistent. Thus, we plan to perform a systematic review to clarify the efficacy and safety of moxibustion in patients with gastric cancer who underwent surgical therapy or chemotherapy by summarizing the currently available RCT evidence.

## 2 Methods

### 2.1 Study registration

This protocol has been registered in the International Prospective Register of Systematic Reviews (PROSPERO) platform (registration number: CRD42020169511). We reported the protocol according to the Preferred Reporting Items for Systematic Review and Meta-Analysis Protocols (PRISMA-P) statement.^[17]^

### 2.2 Inclusion and exclusion criteria

#### 2.2.1 Types of studies

We will include RCTs testing the effects of moxibustion in patients with gastric cancer who underwent surgical therapy or chemotherapy. Both parallel and crossover trials will be eligible. Non-randomized controlled trials, such as preference clinical trials, will be excluded.

#### 2.2.2 Types of participants

We will include patients with gastric cancer diagnosed by any recognized criteria for which histopathological evidence is necessary. The patients should receive any type of surgical therapy (e.g., partial gastrectomy, total gastrectomy, palliative surgery, etc) and/or any regimen of chemotherapy. There are no limitations on age, sex, or stage of gastric cancer. RCTs enrolling patients with non-primary gastric cancer, such as stomach metastases from breast cancer, will be excluded.

#### 2.2.3 Types of interventions

Any kind of moxibustion burning moxa to generate heat stimulation will be eligible, such as direct moxibustion, sandwiched moxibustion, and heat-sensitive moxibustion. Moxibustion can be used alone or in combination with other treatments (e.g., proton pump inhibitors) for treating side effects. Other heat-stimulation therapies not involving the burning of moxa are ineligible, such as infrared laser moxibustion and medicinal moxibustion.

#### 2.2.4 Types of controls

Eligible comparisons will include moxibustion + non-moxibustion treatment vs non-moxibustion treatments or moxibustion alone vs placebo/no treatments. The use of other acupoint or meridian therapies (e.g., acupuncture, acupressure, Tuina, etc.) will not be allowed in either groups.

#### 2.2.5 Types of outcomes

The outcomes are separately defined for the side effects of surgical therapy and chemotherapy.

For analyses of the side effects of both surgical therapy and chemotherapy, the primary outcomes are defined as the incidence of any side effect, response rate assessed by any validated criteria, and quality of life assessed by any validated scale.

For the analyses of the side effects of surgical therapy, the secondary outcomes include the incidence of each individual side effect (e.g., abdominal pain, abdominal distention, and constipation), time to first flatus (hours), time to first defecation (hours), time to first bowel sounds (hours), and length of in-hospital stay (days). For the analysis of the side effects of chemotherapy, the secondary outcomes include the incidence of each individual side effect (e.g., vomiting, fatigue, and myelosuppression), count of white blood cell (10^9^/L), count of red blood cell (10^12^/L), count of platelets (unit: 10^9^/L), and level of hemoglobin (g/L). Moxibustion-related adverse effects will be the safety outcome for both analyses.

### 2.3 Search methods for the identification of studies

#### 2.3.1 Data sources

We will systematically search 3 English databases including PubMed, Embase, the Cochrane Library and four Chinese databases including Sinomed, Chinese National Knowledge Infrastructure, Wanfang, and VIP. The ongoing RCTs will be searched in two trial registry platforms, clinicaltrials.gov and the Chinese Clinical Trial Registry. The time frame of the search is expected to be from the inception of the databases to July 1, 2020. The publication language will not be limited.

#### 2.3.2 Search strategy

The search strategy in PubMed, as an example, will be composed of the following medical subject headings and free words: (stomach neoplasms [mh] OR gastric cancer∗ [tw] OR gastric carcinoma [tw] OR gastric tumor∗ [tw] OR gastric neoplasm∗ [tw] OR stomach cancer∗ [tw] OR stomach carcinoma [tw] OR stomach tumor∗ [tw] OR stomach neoplasm∗ [tw]) AND (moxibustion [mh] OR moxibustion [tw] OR moxa [tw]) NOT (animals [mh] NOT humans [mh]). The search strategy will be adjusted for other databases according to their specific rules. We will also search the references of all previous reviews focusing on similar topics to obtain additional studies for inclusion.

### 2.4 Data collection and analysis

#### 2.4.1 Study selection

**Figure 1:**
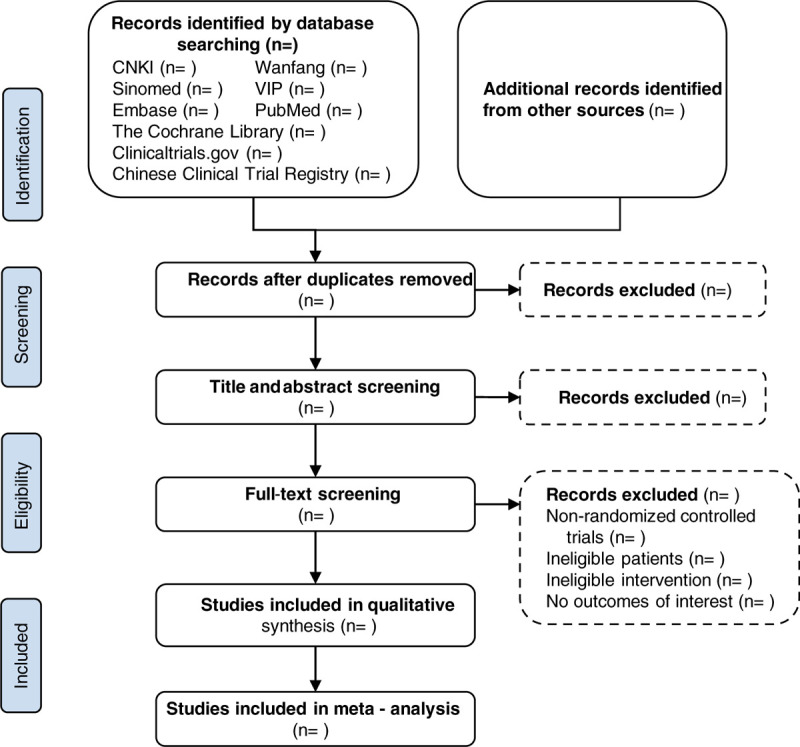
Flowchart of search and screening. CBM = Chinese Biomedical Literature Database, CNKI = China National Knowledge Infrastructure.

The bibliographies yielded by the search will be imported into Endnote X9 (Clarivate Analytics US LLC) software for deduplication. Two reviewers, in duplicate and independently, will first exclude irrelevant literature by reading the titles and abstracts and will subsequently determine final inclusion by reading through the full texts. If there is any inconsistency between the reviewers, they will reach an agreement by discussion or by the judgment of a third reviewer. The detailed process of the literature search and study selection will be presented in a PRISMA-style flowchart (Fig. 1).

#### 2.4.2 Data extraction

Using a pilot-tested electronic form, 2 reviewers, in duplicate and independently, will extract the following key information from each included RCT:

1) study characteristics including the name of the first author and publication time, length of follow-up, and funding;
2) patient characteristics including sex, age, stage of gastric cancer, and course of disease;
3) treatment information including type, acupoints, and dose of moxibustion, type of surgical therapy, regimen of chemotherapy, details of basic treatments, and course of treatment; and
4) outcome-related data: for continuous variables, the means and standard deviations of the characteristics, and final follow-up data will be extracted; for binary variables, events and total number of patients will be extracted. The reviewers will finally cross-check the results, and discrepancies will be resolved by discussion or a third reviewer's decision.

#### 2.4.3 Risk of bias assessment

Working independently and in duplicate, 2 reviewers will assess the risk of bias within each RCT using the Cochrane risk of bias tool.^[18]^ This tool includes seven questions and assesses six domains of the risk of bias:

1) selection bias (random sequence generation method and allocation concealment);
2) performance bias (blinding of patient and clinicians);
3) measurement bias (blinding of outcome evaluators);
4) attrition bias (completeness of result data);
5) reporting bias (selective reporting);
6) other bias.

The reviewers will assess each domain of bias as “low risk”, “unclear risk,” or “high risk” and will resolve any disagreements by consensus or seeking a decision from a third reviewer.

#### 2.4.4 Managing missing data

We will try to request any missing information from the corresponding authors of the RCTs via emails. We will also impute missing standard deviations from the median and full or interquartile range.^[19]^ If the missing information cannot be obtained in these ways, we will exclude the trials from the quantitative analyses.

#### 2.4.5 Data synthesis

The meta-analysis will be separately performed for the outcomes of the side effects of surgical therapy and chemotherapy. The “meta” and “forestplot” packages for R v3.6.2 (Ross Ihaka, Robert Gentlemen, New Zealand) software will be used to perform the meta-analyses and draw forest plots, respectively. We will pool continuous data using the inverse variance method where mean differences or 95% confidence intervals (CIs) will be used as the effect measures. When the units cannot be converted to be unified, we will calculate standardized mean differences and 95% CIs and pool them. For dichotomous outcomes, we will use the relative risks and 95% CIs as the combined effect measures, and the meta-analyses will be performed using the Mantel–Haenszel method. For the ordinal outcomes, we will calculate and pool the proportional odds ratios. A random effects model will be preferred in all meta-analyses.

#### 2.4.6 Assessment of heterogeneity

Cochran's Q test will be used to determine whether there is significant statistical heterogeneity across the included studies, and the I^2^ statistic will also be calculated to quantitatively detect heterogeneity. Heterogeneity will be considered to be small when the *P* value in the Q test > 0.10 and I^2^ < 50%. If the *P* value in the Q test < 0.10 or I^2^ > 50%, the heterogeneity will be considered to be significant. In this case, we will try to explore the sources of heterogeneity by subgroup analyses. If the heterogeneity is clinically unacceptable, we will choose a narrative summary rather than combining the individual study data.

#### 2.4.7 Subgroup analysis

The following subgroup variables with a hypothesized result direction based on our clinical considerations will be tested to identify the source of heterogeneity:

Stage of cancer: no distant metastasis vs distant metastasis; we anticipate that the effect magnitude will be larger in RCTs enrolling patients without distant metastasis;

Type of moxibustion: direct moxibustion vs indirect moxibustion; direct moxibustion is defined as those types of moxibustion where the moxa cone or stick is suspended above the skin's surface (acupoints), mainly including mild moxibustion and heat-sensitive moxibustion, while direct moxibustion refers to those types of moxibustion where the moxa is separated by some materials, including ginger-separated moxibustion, garlic-separated moxibustion, aconite cake-separated moxibustion, long snake moxibustion, etc.; we anticipate that the effect magnitude will be larger in RCTs testing indirect moxibustion;

Course of moxibustion: < 7 days of treatment versus ≥ 7 days of treatment for RCTs of the side effects of surgical therapy; < 4 weeks of treatment vs ≥ 4 weeks of treatment for RCTs of side effects of the chemotherapy; we anticipate the effect magnitude will be larger in RCTs with longer courses of treatment.

#### 2.4.8 Sensitivity analysis

We will evaluate the robustness of the results by excluding RCTs with three or more domains with a high risk of bias.

#### 2.4.9 Assessment of publication bias

For the outcomes of which ten or more RCTs are included, we will judge whether there is significant publication bias across the RCTs by drawing funnel plots and performing Egger tests. Asymmetry observed in the funnel plots or a *P* value less than .05 indicates a significant publication bias.

#### 2.4.10 Assessment of the quality of evidence

The Grading of Recommendations Assessment, Development and Evaluation system^[20]^ will be applied to appraise the quality of evidence for the outcomes. The initial quality of the meta-analytic evidence from RCTs will be high. The reviewer will have the option of downgrading one or two levels of quality of evidence according to the limitations on each risk of bias, indirectness, imprecision, inconsistency, and publication bias. The quality of evidence will finally be determined to be high (no limitations in all aspects), moderate (−1 level), low (−2 levels), or very low (−3 or more levels).

### 2.5 Ethics and dissemination

This systematic review protocol does not involve any intervention or breach of personal privacy; thus, ethical review is not needed. We aim to publish the results in a peer-reviewed journal.

## 3 Discussion

Our preliminary literature review has found that there are dozens of RCTs investigating the efficacy and/or safety of moxibustion for the side effects of surgical therapy or chemotherapy in patients with gastric cancer. Their sample sizes, however, are generally small, so that they cannot provide high-quality evidence with sufficient statistical power, and their conclusions are inconsistent. Two previous systematic reviews (Zhang et al^[21]^ and Huang et al^[22]^) discussed the effects of moxibustion on alleviating chemotherapy-induced side effects, but included only three and one RCTs of gastric cancer. The two previous reviews also did not assess any outcome regarding post-gastrectomy side effects.

In this systematic review, we will include as much RCT evidence as possible to provide a comprehensive analysis of all patient important outcomes. The prespecified subgroup hypotheses could improve the credibility of the subgroup results.^[23]^ A potential limitation of this systematic review is that the precision of the estimates will be naturally affected by the quality of the primary RCTs. Nevertheless, we can expect that this systematic review will provide the current best evidence on moxibustion for the side effects of surgical and medical therapies for gastric cancer.

## Author contributions

**Conceptualization**: Xu Zhou

**Investigation**: Shuqing Li, Jianrong Chen, Yanping Wang

**Funding acquisition**: Xu Zhou

**Supervision**: Weifeng Zhu

**Methodology**: Weifeng Zhu

**Writing – original draft**: Shuqing Li, Jianrong Chen

**Writing – review & editing**: Xu Zhou, Weifeng Zhu
